# Large Preferred Region for Packaging of Bacterial DNA by phiC725A, a Novel *Pseudomonas aeruginosa* F116-Like Bacteriophage

**DOI:** 10.1371/journal.pone.0169684

**Published:** 2017-01-06

**Authors:** Christine Pourcel, Cédric Midoux, Yolande Hauck, Gilles Vergnaud, Libera Latino

**Affiliations:** Institute for Integrative Biology of the Cell (I2BC), CEA, CNRS, Univ. Paris-Sud, Université Paris-Saclay, Gif-sur-Yvette, France; UNITED STATES

## Abstract

Bacteriophage vB_PaeP_PAO1_phiC725A (short name phiC725A) was isolated following mitomycin C induction of C7-25, a clinical *Pseudomonas aeruginosa* strain carrying phiC725A as a prophage. The phiC725A genome sequence shows similarity to F116, a *P*. *aeruginosa* podovirus capable of generalized transduction. Likewise, phiC725A is a podovirus with long tail fibers. PhiC725A was able to lysogenize two additional *P*. *aeruginosa* strains in which it was maintained both as a prophage and in an episomal state. Investigation by deep sequencing showed that bacterial DNA carried inside phage particles originated predominantly from a 700-800kb region, immediately flanking the *attL* prophage insertion site, whether the phages were induced from a lysogen or recovered after infection. This indicates that during productive replication, recombination of phage genomes with the bacterial chromosome at the *att* site occurs occasionally, allowing packaging of adjacent bacterial DNA.

## Introduction

Some bacteriophages are capable of transduction, as shown by their capacity to transfer a DNA fragment from one bacterium to another, resulting in acquisition of new genetic information. As opposed to specialized transduction in which transduction is limited to host DNA adjacent to prophage insertion site [1], generalized transduction (GT) is defined as a process in which any host gene can be transferred to another bacterium. Mu-like phages perform transduction following transposition at multiple sites in the bacterial chromosome, in the course of their replicative cycle [2, 3]. Other transducing phages perform rolling-circle replication and use packaging initiation (*pac*) sites for packaging their genome via a headful mechanism [4]. It has been suggested that cryptic *pac* site on the host chromosome may lead to packaging of bacterial DNA by such phages [5–7].

The most studied phages performing GT are *Escherichia coli* phage P1 [8] and *Salmonella enterica* phage P22 [9]. Phage F116, also capable of GT, was isolated from a *Pseudomonas aeruginosa* clinical strain [10]. It was reported to maintain its DNA as a plasmid [11] and it was not clear whether it could integrate into the bacterial genome [12]. F116 binds to the type IV pili of its host [13] and can digest alginate [14]. Upon sequencing of its genome (65,195bp, genome accession number AY625898), it was shown that 2.6% of the packaged DNA was of bacterial origin, predominantly derived from a particular region of the chromosome [15]. Two F116-like *P*. *aeruginosa* temperate phages have been sequenced, H66 (65,270bp, genome accession number KC262634) and LKA5 (64,746bp, genome accession number KC900378). In addition, several *P*. *aeruginosa* strains were shown to possess a prophage with up to 98% DNA sequence similarity with F116 [16]. Based on genome and protein comparison these phages are recognized as a homology group [17].

Here we describe a novel F116-like phage induced from a clinical *P*. *aeruginosa* strain which, together with previously described phages and prophages, contribute to the forming of a new genus sharing common characteristics. We demonstrate that the phage preferentially packages bacterial DNA localized on one side of its insertion site, not only when recovered by induction of the prophage, but also after an infection cycle.

## Materials and Methods

### Ethics statement

The present project is in compliance with the Helsinki Declaration (Ethical Principles for Medical Research Involving Human Subjects). Bacterial strains were previously collected as part of the patients' usual care, without any additional sampling for the present investigation [18–20]. The ethic committee “Comité Consultatif pour la Protection des Personnes dans la Recherche Biomédicale (CCPPRB) Ile-de-France”, who was consulted, specifically approved the study, and declared that patient informed consent was not needed.

### Strains and media

Strain C7-25 was isolated from a cystic fibrosis patient and was previously investigated as part of a project on antibiotic resistance [20] and on susceptibility to bacteriophages [21]. Its genome has been totally sequenced and is presently under analysis (Pourcel et al. unpublished). PcyII-10 and 26 additional clinical strains were from Percy Hospital, Clamart, France. Phages vB_PaeP_PAO1_Ab05 (Ab05), vB_PaeM_PAO1_Ab17 (Ab17) and vB_PaeM_PAO1_Ab27 (Ab27) were described in [22]. PAO1 LPS and type IV pilus transposon mutants were obtained from “The *P*. *aeruginosa* Transposon Mutant Library”, UW Genome Sciences, USA. Luria broth (LB) medium supplemented with 2 mM CaCl_2_ was used for bacterial growth and phage titration. Saline magnesium (SM) buffer (50 mM Tris-HCl pH7.5, 100 mM NaCl, 8.1 mM MgSO_4_, 0.01% gelatin) was used to preserve purified phages at 4°C.

### Phage production

To induce bacteriophages from C7-25, 20 ml LB medium were inoculated at 1/100 with an overnight C7-25 culture and shaken at 37°C until the culture reached an A_600_ of 0.6. Mitomycin C was added to a final concentration of three μg ml^−1^ and the incubation was continued until lysis occurred. Culture supernatant was tested for the presence of bacteriophages by plating five μl of different dilutions after infection of a PAO1 strain culture. A single plaque was recovered and purified by three successive platings. The newly isolated phage phiC725A was amplified for 8 hours on solid medium, by mixing 10^9^ cfu of PAO1 with 10^6^ pfu of phiC725A per plate, and collected as previously described [23]. Bacteria and debris were pelleted by centrifugation, the supernatant was recovered and phages were precipitated with 10% polyethylene glycol (PEG) 8000 overnight at 4°C. After centrifugation at 15,000xg for 20 min, the pellet was suspended in one ml of phosphate-buffered saline (PBS) and treated for two hours with DNase I (50 μg ml^−1^) at 37°C. Three chloroform extractions were performed, prior to filtration through a 0.45μM filter and centrifugation for two hours at 260,000xg. The pellet was suspended in 50 μl of PBS. For electron microscopy (EM) visualization, five μl of phage suspension were stained with 2% potassium phosphotungstate (pH 7.0) as previously described [21].

### DNA extraction and PCR

Samples were lysed in lysis buffer (Tris 10 mM, pH 7.8, EDTA 10 mM, NaCl 10 mM, SDS 0.5%wt/vol), treated with proteinase K at 50 μg ml^-1^ for 2 hours at 50°C, followed by one phenol and one chloroform extraction, and ethanol precipitation.

Prophage insertion was detected by PCR performed on purified DNA. Amplification using primer pairs C725-Ins-F 5’TGCCGGACGTTCCGGCTTCA3’ and C725-Ins-R 5’CGATGTTTTACCGCAAGTCG3’ resulted in an amplification product if a prophage was inserted at the bacterial *att* site (*attB*). Amplification using primer pair phi725-Reg2-F 5’GAATTGTGGCGGAAGCCAAC3’ and phi725-Reg2-R 5’GCAATAGCAGTTTCTGCGTG3’ present inside the phage genome on both sides of the phage attachment site (*attP)* was possible if an intact *attP* site was present, as in non-integrated phage genomes.

### Whole genome sequencing

Two μg of purified phage or bacterial DNA were sent for draft whole genome sequencing to the IMAGIF MiSeq Illumina platform (CNRS, Gif sur Yvette, France). Libraries were made from sheared fragments of DNA (average size 900bp), and 250bp or 300 bp paired-end reads were produced. Phage genome assemblies, sequencing reads mapping to genome reference, prophage insertion sites and other sequence analyses were done using tools in Geneious R9 (Biomatters, New Zealand).

### Nucleotide sequence accession numbers

The DNA sequence of phage phiC725A has been deposited in the EMBL-EBI database under accession number LT603684 within project PRJEB14922. Sequencing reads from the phage induction and infection experiments as well as sequencing reads from the PAO1_Or_ lysogen for phiC725A were also deposited in project PRJEB14922 and are available from the European Nucleotide Archives (ENA) browser at http://www.ebi.ac.uk/ena/data/view/PRJEB14922.

## Results

### Isolation of phiC725A

**Fig 1:**
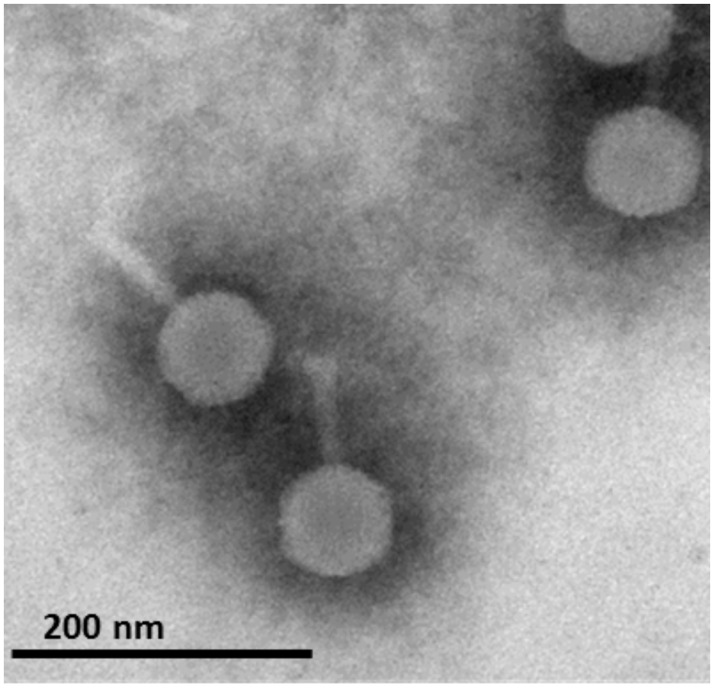
Electron microscopy observation of phage phiC725A. The black bar represents 200nm.

We observed that strain C7-25 spontaneously released phages, as evidenced by the presence of small plaques on a lawn of bacteria after several days of growth on solid LB medium. Upon treatment of planktonic cells with mitomycin C, bacterial growth was rapidly stopped and massive lysis was observed. A bacteriophage forming small clear plaques with a dark center was isolated from the culture supernatant and amplified on a reference strain PAO1 representative called PAO1_Or_ (the genome sequence of the PAO1 representative used in our laboratory in Orsay (Or) was previously reported [24]). Upon examination by EM, the phages displayed a large head (78nm), and what seemed to be a short thin tail (66nm) (Fig 1). No evidence of tail flexibility or contractility could be seen from EM observation of numerous phage particles. A similar morphology was previously reported for phage F116 and it was recently proposed to classify F116 as a podovirus with tightly packed long fibers [15, 25, 26]. The new phage was called vB_PaeP_PAO1_phiC725A (short name phiC725A), for prophage A of strain C7-25. The phage host range was tested by infecting 26 clinical strains, including the reference strains PAO1 (PAO1_Or_) and PA14. Two were susceptible (PAO1 and PcyII-10) and six showed a reduced efficiency of plating. The rest were totally resistant. In order to identify the receptor for phage phiC725A, different amounts of phages (from 10^4^ to 10^7^ pfu) were spotted onto PAO1 transposon mutants affected in type IV pilus genes (*pilY1*, *pilR*, *pilA* and *pilQ*) and LPS synthesis genes (*wzy*, *algC*). No growth was observed on mutants lacking pili whereas normal growth was seen on LPS mutants.

### The phiC725A phage genome

**Fig 2:**
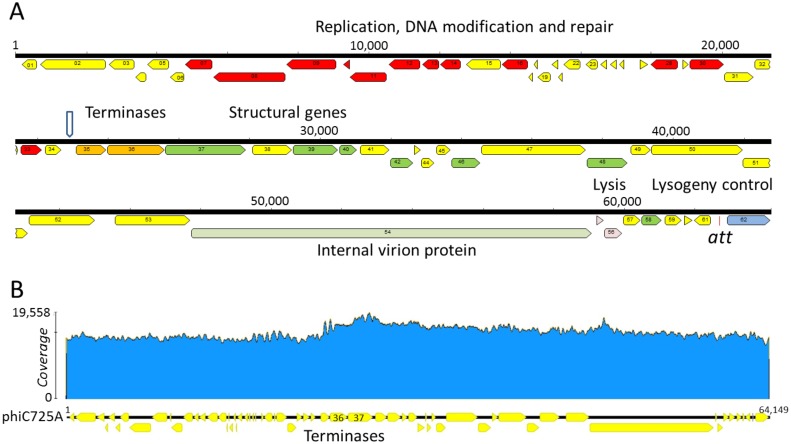
Genomic organization of phiC725A. A) Distribution of the predicted CDSs along the phage genome. CDSs are shown as horizontal arrows. Position 1 in the sequence is defined in keeping with phage F116. The different colors correspond to the putative function: red, DNA replication, modification and repair; orange, terminase; green, morphogenesis and packaging; light green, putative RNA polymerase; pink, lysis; blue, lysogeny control; yellow, unidentified. The vertical arrow indicates the putative localization of the *pac* site. The *att* site towards the right end is in the TCTCTCCGTCCGCACCA orientation, i.e. the reverse orientation with respect to the PAO1Or host sequence orientation. B) Mapping of the sequencing reads showing an excess at the level of the terminase subunits genes, with a progressive decrease towards the right end.

PhiC725A was amplified on PAO1_Or_, then phage particles were purified and treated with DNAse I before extracting the DNA for sequencing. To be able to characterize the phage genome but also the bacterial DNA packaged within the phage head, deep sequencing of the phage genome was performed so that an average coverage of almost 15,000X, was achieved. A total of 1,284,508 reads were obtained, of which 0.6% (7,844) mapped to PAO1_Or_. The phage reads were assembled resulting in a single 65,149bp long molecule with a 63.5% GC content. BLAST analysis on Genbank (release 214) identified F116 (genome accession number AY625898.1) as the closest phage genome with a 86% mean similarity over the whole sequence. PhiC725A showed 84% and 76.5% similarity with phage LK5 (KC900378.1) and H66 (KC262634.1) respectively. Similarities were also found along the genomes of prophages of *P*. *aeruginosa* strains N01-01092 (81.8%, CP012901.1), DHS01 (76.5%, CP013993.1) and H27930 (72.7%, CP008860.1). In keeping with the reference phage F116 genetic map, the first nucleotide was assigned as the first nucleotide downstream the *int* gene (Fig 2A). Annotation led to the identification of 62 putative coding sequences (CDSs) (S1 Table) globally matching those of F116, H66 and LKA5 with some remarkable differences which are most likely the result of recombination events, as shown by alignment of the four phage genomes (S1 Fig). The region encompassing phiC725A_01 to phiC725A_09 showed the highest level of dissimilarity (S1 Fig). PhiC725A_54, a very large protein had a homologue in H66, LKA5 and the three prophages and corresponded to F116p59 and F116p60. In phage H66 this protein is described as a structural protein. By homology search it was shown to possess a domain matching within the internal virion protein D (IVP-D) of *Pseudoalteromonas* phage ΦRIO-1, corresponding to the C-terminal domain of IVP N4 gp50, a very large multisubunits enzyme [17]. N4 gp50 is an RNA polymerase which central part holds the transcriptionally active domain [27]. Such proteins, injected into the host together with the viral DNA, transcribe early genes [28, 29]. In the central part of phiC725A_54 lies a domain matching to the S-adenosyl methionine binding site of DNA methylases. PhiC725A and LKA5 lacked a short CDS (F116p41) present in the F116 genome in between the portal gene (phiC725A_37) and a putative capsid gene (phiC725A_38). PhiC725A, like F116, possesses genes that are common to many podoviruses [17]. Some are characteristic of T7-like podoviruses with tubular tails, but the tail fibers of F116-like phages, as visualized by electron microscopy seem to be longer and suppler.

A 17bp attachment site (*attP*: TGGTGCGGACGGAGAGA) was identified at position 62,750 upstream of the integrase of phiC725A and was identical to a sequence in between tRNA-His and tRNA-Leu in PAO1 (*attB* in position 1,947,646–1,947,662 of PAO1 accession number AE004091, and 3,568,180 to 3,568,196 of PAO1_Or_ accession number LN871187.1). It corresponded to the site of the prophage insertion in strain C7-25 (position 3,680,043–3,744,208 in the C7-25 genome, Pourcel et al. unpublished). The same insertion site was observed for F116-like prophages in *P*. *aeruginosa* strains N01-01092 and H27930. In contrast, in strain DHS01 (accession number CP013993.1) the prophage was present at position 2,550,071–2,615,626 corresponding to 2,363,267–2,363,274 of PAO1_Or_ (accession number LN871187.1). The insertion was flanked by a 11 bp direct repeat sequence TCCATCATCGG. F116 has been described as a non-integrative phage [11], but it possesses a putative integrase, identical to that of phiC725A (F116p70) and the TGGTGCGGACGGAGAGA *attP* site, suggesting that this phage could in theory lysogenize strain PAO1. The 17 bp *attB* site sequence identified here was present in 93 out of the 95 *P*. *aeruginosa* complete genome sequences which could be queried in Genbank (the site was absent in strains ATCC 27853 and Cu1510).

When the full dataset of phage reads was mapped back on the phiC725A sequence (Fig 2B), no high peak of reads characteristic of fixed genome ends was observed [22]. This is as expected if the phage genome is terminally redundant (TR) and circularly permuted (CP), as previously demonstrated for F116 [30]. However, we noticed a maximum in the coverage starting at position 23,500 (near the small and large terminase subunit genes) and decreasing over the whole genome (Fig 2B). This behavior might be related to the phage replication or recombination, which leads to the generation of free ends. It could also indicate the position of the packaging initiation of the phage genome, with no clear-cut starting point, i.e. a lack of specificity in cleavage initiation. Indeed there are numerous examples of *pac* sites residing in or near the terminase genes in different phage genomes [31, 32]. Because of the terminal redundancy, a portion of the genome starting from the packaging position will be overrepresented.

### Bacterial DNA packaged into phiC725A virions

We then examined the 0.6% of host genome reads obtained from sequencing purified bacteriophages. Surprisingly, we found that 73% (5717 among 7844 bacterial reads) mapped over a region corresponding to the *attB* site, with a maximum coverage at the *attB* position and extending over 700–800 kb on one side only of the *attB* site (S2 Fig). The remaining reads of bacterial origin were homogeneously distributed along the whole genome. We detected six hybrid reads containing a *attP/B* site, suggesting that during phage replication some genomes inserted within the bacterial chromosome at *attB* site, leading to packaging of adjacent DNA.

**Fig 3:**
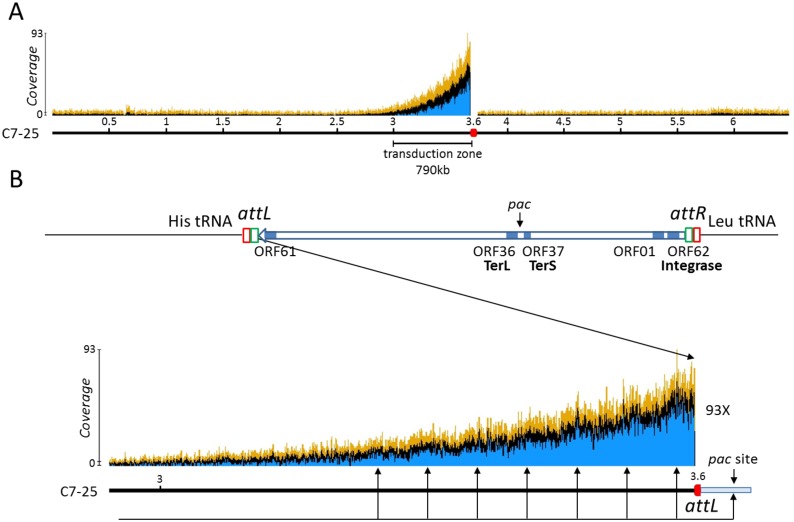
Distribution of packaged bacterial reads from C7-25 mitomycin C-induced phages. A) Mapping of the bacterial reads along the PAO1_Or_ genome. The blue horizontal arrow represents the prophage. B) Organization of the phiC725A prophage and close-up of the 800kb left flanking region; the distribution of the packaged bacterial sequence reads is shown. The collection of arrows underneath the graph and starting from the right at the tentative prophage *pac* site within the blue bar representing the phage genome, correspond to coverage maximums. The first such maximum is located approximately 25 kb from the left of the blue bar. The spacing between the arrows is approximately 72kb, compared to the 65 kb of phage genome suggesting that the terminal redundancy represents 10% of the phage size. The blue, black and yellow lines represent the minimum, average and maximum read coverage observed respectively.

We consequently decided to analyse the phages directly purified following mitomycin C induction of strain C7-25. Bacteriophages were treated with DNAse I to eliminate contaminating bacterial DNA, before lysis and extraction of DNA. Sequencing of 1,834,389 DNA fragments with an average fragment size of 680 bp, resulted in the production of 3,668,778 sequence reads. Most reads corresponded to phage sequence, achieving an average coverage of the phage genome of more than 25,000X. However, 81,578 reads, or 2.2% of the total amount or reads, corresponded to the bacterial genome. 55,000 reads of these (67%) mapped over the 700–800 kb region on the left side of the prophage insertion *att* site with a regular decay, similarly to what was observed during infection (Fig 3A). The average coverage was 60-70X in the vicinity of the *attL* site and was down to 1.8X, 790 kb away. The remaining 26,578 reads of bacterial origin were distributed along the whole genome achieving a mean coverage of 1.3X. On close examination of the reads distribution (Fig 3B), it appeared that it formed waves of about 65-75kb, slightly larger than the size of the phage genome.

More than 25,000 sequence reads contained the *att* sequence in agreement with the phage genome coverage. In the vast majority of cases, it was the *attP* from *bona fide* encapsidated phage genomes, but fifty-six reads contained an *attP/B site*. Only hybrids corresponding to the left side of the *attB* site (*attL*) were recovered, showing that bacterial DNA packaging at this location was unidirectional and initiated within a copy of the phage genome integrated at the *attB* site (Fig 3B).

Consequently, the majority of transduced bacterial DNA appears to be associated with the encapsidation of phage linked with the *attB* site in both the infection and induction experiments. The only detectable difference is a three-fold higher ratio of packaged host DNA in the induction of strain C7-25 (2.2% +/- 0.015 (P<0.05) versus 0.6% +/- 0.014 (P<0.05) for infection of strain PAO1. These differences are highly significant (X^2^ test p-value < 2.2e-16).

### Production of lysogens using phage phiC725A

PhiC725A was able to lysogenize both PAO1_Or_ (PAO1_phiC725A_) and PcyII-10 (PcyII-10_phiC725A_) as shown by the stable presence of the phage DNA tested by PCR, continuous production of phages (10^8^ pfu ml^-1^ in the culture supernatant), and exclusion of super-infection by phage phiC725A. In order to test for heteroimmunity, the lysogens were challenged with three different phages which grew on the parental PAO1_Or_ and PcyII-10 strains. Ab05, a phi-KMV-like phage which uses type IV pili as receptor, produced plaques on the PAO1_phiC725A_ lysogen, although of smaller size, and grew poorly on the PcyII-10_phiC725A_ lysogen. Ab17 and Ab27, which use LPS as receptors, grew normally. This suggested that phiC725A lysogens were not heteroimmune but restricted infection by phages using pili as a receptor.

To check whether the phage was inserted into the bacterial genome, we performed a PCR reaction for *attP/B* with primer pair C725-Ins containing one primer flanking the *att* site on one side in the bacterial genome and one primer on the other side inside the phage genome. A signal was obtained with the C7-25 strain used as positive control and with both PAO1_phiC725A_ and PcyII-10_phiC725A_ indicating that the prophage integrated at the same *attB* site (data not shown). The (control) parent strains PAO1_Or_ and PcyII-10 were negative. A PCR reaction with phage primers localized on both sides of *attP*, aiming at detecting non-integrated genomes (primer pair Phi725-Reg2) was also positive with all three lysogens, indicating that free viral genomes were present in the cells (data not shown).

## Discussion

We describe phiC725A, a novel F116-like virus of *P*. *aeruginosa*, and provide evidence that the phage can integrate its genome at a specific site in a tRNA genes locus, and also remains as an episome. Lysogens continuously produce phages. In both prophage induction and infection, we show that phiC725A packages bacterial host DNA originating preferentially from one side of the *attB* site, and expanding over several hundreds of kilobases. This portion of the genome does not correspond to a region of high sequence diversity as described by Spencer at al. [33], but is one of the region of genomic plasticity (RGP) described by Mathee et al. [34]. Indeed at this position a phage was found inserted in *P*. *aeruginosa* strain PA2192. It is known that tRNA genes often serve as insertion sites for phages and other integrative elements. Genes present in the 800kb region preferentially packaged within phages are supposedly involved in different metabolic functions and some might play a role in virulence. Immediately flanking the *attB* site is the his-tRNA, then the *parRS* operon, encoding the two-component regulatory system ParR-ParS, involved in adaptive resistance to polymyxin B and colistin [35]. A gene encoding a membrane-bound lytic murein transglycosylase (mltD), a virulence-related factor that may participate in beta-lactam resistance is located a few kilobases away [36].

Importantly, we also show that packaging of this region is associated with the presence of a phage genome inserted at the *att* site as demonstrated by the existence of hybrid reads. It appears that packaging of bacterial DNA at the *attL* site takes place during phage replication but is three times less frequent during infection as compared to activation of a prophage. A similar and fully compatible observation of a biased origin of packaged host DNA was made during sequencing of F116, but the extent of bacterial chromosome and the distribution of reads could not be mapped with such precision [15]. In particular, the link with the *att* site and the evidence for the occasional integration of phage genome could not be detected with the 4X phage genome coverage achieved at that time, and the authors instead hypothesized that the bias might result from the presence of one or more cryptic *pac* sites in this region. Massively parallel sequencing used here allowed to achieve the much higher coverage necessary for the finding of hybrid phage-bacteria sequences, demonstrating the role of integrations in the *att* site. Its close similarity to F116 suggests that phiC725A is likely to replicate by rolling circle amplification of circular phage genome. It is expected that virions encapsidate phage DNA from these concatemers using a headful packaging strategy, therefore producing circularly permuted genomes with a terminal redundancy [37, 38]. A *pac* site inside the phage provides the first cut, and headful DNA packaging is then performed. In phages P22 and SPP1, the small terminase subunit TerS binds to the *pac* site and recruits TerL that cleaves the DNA, initiating packaging [4, 32]. Packaging of host DNA flanking a P22 excision-defective prophage on one side was reported by Youderian et al. [39, 40]. In several temperate phages found in *Staphylococcus aureus* strain RF122, Moon *et al*. showed that TerS and TerL proteins may bind to the *pac* site of the prophage and initiate packaging of the phage DNA and adjacent DNA [41]. This resulted in the mobilization of a genomic island by the temperate bacteriophage. Huang *et al*. inserted *pac* sites in the *E*. *coli* chromosome and showed that P1 packaging of bacterial genes to one side of and near to a *pac* site was increased more than 10-fold. This effect diminished with distance but packaging could be detected over 30% of the length of the chromosome [5].

The sequence data presented here indicates that occasionally, during replication and expression of late genes, phage genome recombines with the bacterial host genome at the *attB* site thus providing a *pac* site. This happens following activation of a prophage in a lysogen, as well as during the lytic cycle in an infected bacteria, although at a lower frequency. The *pac* recognition event will produce one virion containing part of the phage genome together with bacterial genome flanking the *att* site, followed by up to ten-twelve virions (800 kb equivalent taking into account 10% terminal redundancy) containing bacterial DNA (Fig 3B). The absence of clear-cut boundaries suggests that there is no specific cut during packaging. The decay profile of bacterial DNA coverage further indicates that, on average, one event of packaging from an integrated phage will result in three virions containing bacterial DNA. It is also possible that a concatemer of phage DNA is formed at the *attB* site, or more likely sometimes recombines with the *attB* site after replication and prior to packaging. As a consequence, the population of hybrid virions, i.e. containing phage DNA and bacterial DNA, as well as the extent of encapsidation of bacterial genome, will be heterogeneous. If many phage genomes are attached downstream the packaging site, no or only few “bacterial” virions will be produced and vice-versa.

## Conclusion

PhiC725A is a novel F116-like temperate phage capable of transporting bacterial DNA at a high frequency. We show for the first time that bacterial DNA is packaged preferentially from one side of the prophage integration site not only during prophage induction but also during infection, indicating that packaging can occasionally be initiated in a prophage-like configuration and will then extend to neighboring DNA. This implies that general transduction by phiC725A is highly biased which may have interesting consequences in terms of *P*. *aeruginosa* genome evolution.

## Supporting Information

S1 Fig Alignment of four F116-like phage genomes; phiC725A, F116, LKA5, H66 using the Geneious software. (TIFF)

S2 Fig Distribution of packaged bacterial reads from PAO1_Or_ infected by phiC725A. Mapping of the bacterial reads along the PAO1_Or_ genome. The blue, black and yellow represent the minimum, average and maximum read coverage observed respectively. The read box represents the phage *att* site.
(TIFF)

S1 Table Putative function of CDS observed in annotated phiC725A phage genome. (DOCX)
