# Supplementary figures and images for: Large Preferred Region for Packaging of Bacterial DNA by phiC725A, a Novel Pseudomonas aeruginosa F116-Like Bacteriophage
Source: PLoS One. 2017 Jan 6;12(1):e0169684. doi: 10.1371/journal.pone.0169684 (PMC5217972; doi:10.1371/journal.pone.0169684)

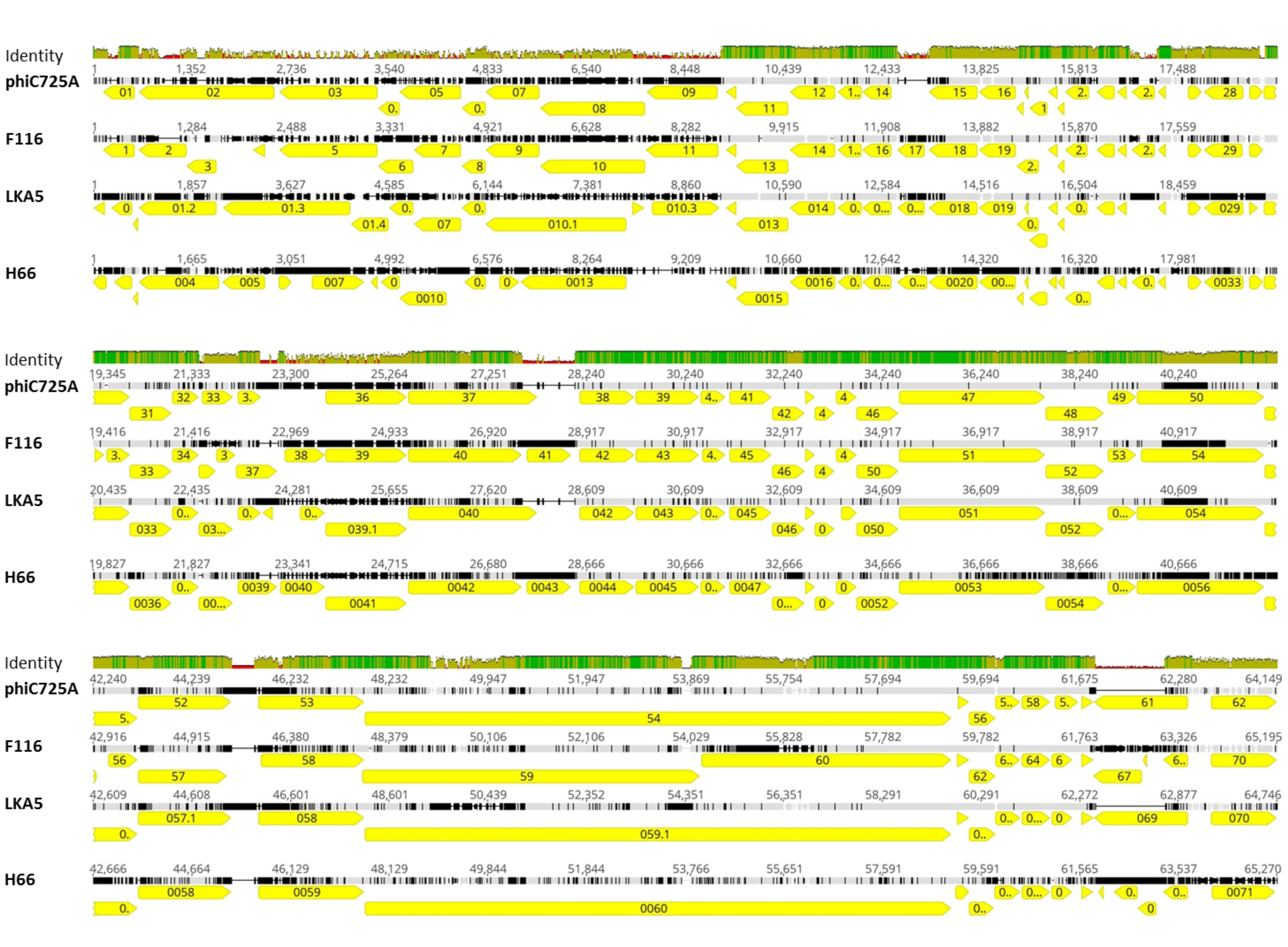

Supplement: S1 Fig — (TIFF) [file pone.0169684.s001.tiff]

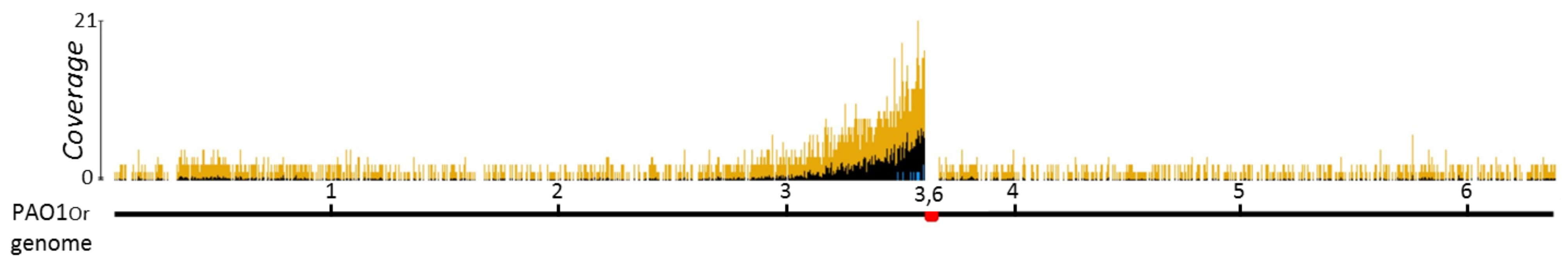

Supplement: S2 Fig — Mapping of the bacterial reads along the PAO1Or genome. The blue, black and yellow represent the minimum, average and maximum read coverage observed respectively. The read box represents the phage att site. (TIFF) [file pone.0169684.s002.tiff]
