# Supplementary material for: Large Preferred Region for Packaging of Bacterial DNA by phiC725A, a Novel Pseudomonas aeruginosa F116-Like Bacteriophage
Source: PLoS One. 2017 Jan 6;12(1):e0169684. doi: 10.1371/journal.pone.0169684 (PMC5217972; doi:10.1371/journal.pone.0169684)
Supplement: S1 Table — (DOCX) [file pone.0169684.s003.docx]

**Table S1** : Annotation of Pf725A genome.

| CDS | Start | End | Length (bp) | Direction | gene | Product |
| --- | --- | --- | --- | --- | --- | --- |
| PF725A_0001 | 186 | 656 | 471 | R |  | hypothetical protein |
| PF725A_0002 | 720 | 2594 | 1875 | R |  | hypothetical protein |
| PF725A_0003 | 2656 | 3414 | 759 | R |  | hypothetical protein |
| PF725A_0004 | 3411 | 3734 | 324 | R |  | hypothetical protein |
| PF725A_0005 | 3731 | 4378 | 648 | R |  | hypothetical protein |
| PF725A_0006 | 4375 | 4818 | 444 | R |  | hypothetical protein |
| PF725A_0007 | 4815 | 5621 | 807 | R |  | DNA adenine methyltransferase |
| PF725A_0008 | 5618 | 7681 | 2064 | R |  | DNA cytosine methyltransferase |
| PF725A_0009 | 7695 | 9125 | 1431 | R | DndC | phosphosulfate reductase |
| PF725A_0010 | 9286 | 9495 | 210 | R | LexA | LexA repressor |
| PF725A_0011 | 9492 | 10547 | 1056 | R | yejK | Nucleoid-associated protein |
| PF725A_0012 | 10574 | 11500 | 927 | R | RdgC | recombinase RdgC exonuclease |
| PF725A_0013 | 11527 | 12030 | 504 | R | ssb | Single stranded DNA-binding protein |
| PF725A_0014 | 12034 | 12639 | 606 | R | polC | DNA polymerase III subunit epsilon |
| PF725A_0015 | 12769 | 13785 | 1017 | R |  | hypothetical protein |
| PF725A_0016 | 13790 | 14545 | 756 | R |  | putative DNA repair protein |
| PF725A_0017 | 14542 | 14694 | 153 | R |  | hypothetical protein |
| PF725A_0018 | 14691 | 14810 | 120 | R |  | hypothetical protein |
| PF725A_0019 | 14807 | 15178 | 372 | R |  | hypothetical protein |
| PF725A_0020 | 15175 | 15396 | 222 | R |  | hypothetical protein |
| PF725A_0021 | 15380 | 15526 | 147 | R |  | hypothetical protein |
| PF725A_0022 | 15526 | 16029 | 504 | R |  | hypothetical protein |
| PF725A_0023 | 16157 | 16528 | 372 | R |  | putative carbon storage regulator protein |
| PF725A_0024 | 16563 | 16775 | 213 | R |  | hypothetical protein |
| PF725A_0025 | 16838 | 17053 | 216 | R |  | hypothetical protein |
| PF725A_0026 | 17097 | 17270 | 174 | R |  | hypothetical protein |
| PF725A_0027 | 17682 | 17948 | 267 | F |  | hypothetical protein |
| PF725A_0028 | 17985 | 18788 | 804 | R | prtR | transcriptional regulator |
| PF725A_0029 | 18890 | 19090 | 201 | F |  | Cro-like protein |
| PF725A_0030 | 19092 | 20096 | 1005 | F |  | putative replication protein |
| PF725A_0031 | 20083 | 20940 | 858 | F |  | hypothetical protein |
| PF725A_0032 | 20933 | 21484 | 552 | F |  | hypothetical protein |
| PF725A_0033 | 21540 | 22154 | 615 | F | ninG | putative NinG family protein |
| PF725A_0034 | 22233 | 22727 | 495 | F |  | hypothetical protein |
| PF725A_0035 | 23107 | 23997 | 891 | F |  | putative terminase small subunit protein |
| PF725A_0036 | 24001 | 25632 | 1632 | F |  | putative terminase large subunit protein |
| PF725A_0037 | 25643 | 27958 | 2316 | F |  | putative portal protein |
| PF725A_0038 | 28105 | 29235 | 1131 | F |  | hypothetical protein |
| PF725A_0039 | 29248 | 30540 | 1293 | F |  | putative major capsid protein |
| PF725A_0040 | 30554 | 31078 | 525 | F |  | putative structural protein |
| PF725A_0041 | 31143 | 32015 | 873 | F |  | RyR domain protein |
| PF725A_0042 | 32012 | 32689 | 678 | F |  | putative structural protein |
| PF725A_0043 | 32686 | 32892 | 207 | F |  | hypothetical protein |
| PF725A_0044 | 32873 | 33280 | 408 | F |  | hypothetical protein |
| PF725A_0045 | 33304 | 33729 | 426 | F |  | hypothetical protein |
| PF725A_0046 | 33729 | 34586 | 858 | F |  | putative structural protein |
| PF725A_0047 | 34589 | 37552 | 2964 | F |  | hypothetical protein |
| PF725A_0048 | 37552 | 38742 | 1191 | F |  | putative structural protein |
| PF725A_0049 | 38810 | 39400 | 591 | F |  | hypothetical protein |
| PF725A_0050 | 39405 | 42002 | 2598 | F |  | hypothetical protein |
| PF725A_0051 | 41995 | 43158 | 1164 | F |  | hypothetical protein |
| PF725A_0052 | 43159 | 45051 | 1893 | F |  | hypothetical protein |
| PF725A_0053 | 45586 | 47748 | 2163 | F |  | hypothetical protein |
| PF725A_0054 | 47748 | 59111 | 11364 | F |  | putative internal virion RNA polymerase |
| PF725A_0055 | 59224 | 59463 | 240 | F |  | putative holin protein |
| PF725A_0056 | 59447 | 59980 | 534 | F |  | putative lysozyme |
| PF725A_0057 | 59977 | 60495 | 519 | F |  | hypothetical protein |
| PF725A_0058 | 60506 | 61099 | 594 | F |  | putative tail fiber protein |
| PF725A_0059 | 61175 | 61672 | 498 | F |  | hypothetical protein |
| PF725A_0060 | 61711 | 61977 | 267 | F |  | hypothetical protein |
| PF725A_0061 | 61981 | 62493 | 513 | R |  | hypothetical protein |
| PF725A_0062 | 62914 | 64149 | 1236 | F |  | putative integrase |
